# Empagliflozin protects the heart against ischemia/reperfusion-induced sudden cardiac death

**DOI:** 10.1186/s12933-021-01392-6

**Published:** 2021-10-04

**Authors:** Zhaoyang Hu, Feng Ju, Lei Du, Geoffrey W. Abbott

**Affiliations:** 1 Laboratory of Anesthesia and Critical Care Medicine, National-Local Joint Engineering Research Centre of Translational Medicine of Anesthesiology and National Clinical Research Center for Geriatrics, West China Hospital of Sichuan University, Chengdu, Sichuan China; 2 Department of Anesthesiology, West China Hospital of Sichuan University, Chengdu, Sichuan China; 3 Bioelectricity Laboratory, Department of Physiology and Biophysics, School of Medicine, University of California, Irvine, CA USA

**Keywords:** Arrhythmia, Ischemia–reperfusion injury, Sodium–glucose co-transporter 2 (SGLT-2) inhibitors, Empagliflozin, ERK1/2

## Abstract

**Background:**

Empagliflozin is a selective sodium–glucose cotransporter 2 (SGLT2) inhibitor used to lower blood sugar in adults with type 2 diabetes. Empagliflozin also exerts cardioprotective effects independent from glucose control, but its benefits on arrhythmogenesis and sudden cardiac death are not known. The purpose of this study was to examine the effect of empagliflozin on myocardial ischemia/reperfusion-provoked cardiac arrhythmia and sudden cardiac death in vivo.

**Methods:**

Male Sprague Dawley rats were randomly assigned to sham-operated, control or empagliflozin groups. All except for the sham-operated rats were subjected to 5-min left main coronary artery ligation followed by 20-min reperfusion. A standard limb lead II electrocardiogram was continuously measured throughout the experiment. Coronary artery reperfusion-induced ventricular arrhythmogenesis and empagliflozin therapy were evaluated. The hearts were used for protein phosphorylation analysis and immunohistological assessment.

**Results:**

Empagliflozin did not alter baseline cardiac normal conduction activity. However, empagliflozin eliminated myocardial vulnerability to sudden cardiac death (from 69.2% mortality rate in the control group to 0% in the empagliflozin group) and reduced the susceptibility to reperfusion-induced arrhythmias post I/R injury. Empagliflozin increased phosphorylation of cardiac ERK1/2 after reperfusion injury. Furthermore, inhibition of ERK1/2 using U0126 abolished the anti-arrhythmic action of empagliflozin and ERK1/2 phosphorylation.

**Conclusions:**

Pretreatment with empagliflozin protects the heart from subsequent severe lethal ventricular arrhythmia induced by myocardial ischemia and reperfusion injury. These protective benefits may occur as a consequence of activation of the ERK1/2-dependent cell-survival signaling pathway in a glucose-independent manner.

## Background

Sudden cardiac death (SCD) is one of the most common causes of mortality worldwide, and ischemic heart disease remains the dominant cause of SCD. To essentially resuscitate the ischemic myocardium, beneficial coronary artery revascularization strategies, such as pharmacological thrombolysis, angioplasty, coronary artery bypass grafting (CABG), and percutaneous coronary intervention (PCI) can effectively restore myocardial blood reperfusion. However, the sudden restoration of oxygenated blood flow may also lead to subsequent severe ischemia/reperfusion (I/R) injury and provoke lethal arrhythmias [1]. Lethal reperfusion injury is a major limitation to the efficacy of current reperfusion therapy, and it was previously found that reperfusion can cause a higher incidence of ventricular fibrillation than coronary artery occlusion [1]. Therefore, there remains an unmet need in developing safe and effective anti-arrhythmic strategies to enhance the tolerance of the heart to ischemia/reperfusion for individuals suffering from ischemic heart disease.

Sodium–glucose co-transporter 2 (SGLT2) inhibitors are licensed for the treatment of type 2 diabetes mellitus (T2DM); however, emerging evidence has also confirmed their powerful therapeutic effect against heart failure. The EMPA-REG OUTCOME trial, including over 7000 diabetic patients with cardiovascular diseases, showed that empagliflozin, a SGLT-2 inhibitor, reduced risk of death from cardiovascular causes, and decreased risk of hospitalization for heart failure [2]. More importantly, subsequent analysis of the EMPA-REG OUTCOME data revealed that the beneficial effect offered by empagliflozin was independent of glycemic control [3]. Thereafter, preclinical experimental studies were conducted to evaluate the potential role of empagliflozin on myocardial salvage. Empagliflozin was found to attenuate doxorubicin-induced cardiac toxicity and improve LV function in non-diabetic mice [4]. Empagliflozin also improved both endothelial function and cardiac remodeling in the metabolic syndrome ZSF1 rat model [5], and improved hemodynamics and attenuated cardiac fibrosis in a hypertensive heart failure rat model [6]. However, despite its known benefits on the heart, the anti-arrhythmic effects of empagliflozin against cardiac I/R injury are poorly characterized. Notably, others have shown that empagliflozin may exert certain infarct-sparing effects in the setting of myocardial ischemia-reperfusion [7] and alleviate atrial remodeling and reduce atrial electrical conduction heterogeneity in diabetic rats [8]. Thus, while no direct evidence has to our knowledge been hitherto presented to show that SGLT2 inhibitors, including empagliflozin, influence arrhythmogenesis during coronary artery disease progression or therapy, there is a possible link between empagliflozin and cardioprotective effects attenuating ischemia/reperfusion-induced ventricular conduction disturbances. Thus, the primary aim of this study was to evaluate whether empagliflozin treatment can reduce ventricular arrhythmogenesis and susceptibility to SCD.

Various signaling pathways have been implicated in cardioprotection, including the pro-survival reperfusion injury salvage kinase (RISK) pathway, and the JAK-STAT pathway. The protective effects of these pathways during reperfusion have been identified [9]. Cardioprotective drugs or strategies, such as volatile anesthetics, ischemic preconditioning and postconditioning can limit myocardial infarct size and prompt cell survival post I/R injury via phosphorylation of signaling molecules in these pathways.

The aims of the present study were first to determine whether empagliflozin treatment can protect the heart and reduce predisposition to sudden cardiac death induced by myocardial ischemia-reperfusion injury during acute myocardial infarction. Second, we aimed to identify the potential role of reperfusion-related signaling pathways in anti-arrhythmic activity afforded by empagliflozin, to potentially provide a basis for the identification of novel strategies for targeted prevention of ischemia-provoked arrhythmias.

## Materials and methods

### Experimental animals

**Fig. 1:**
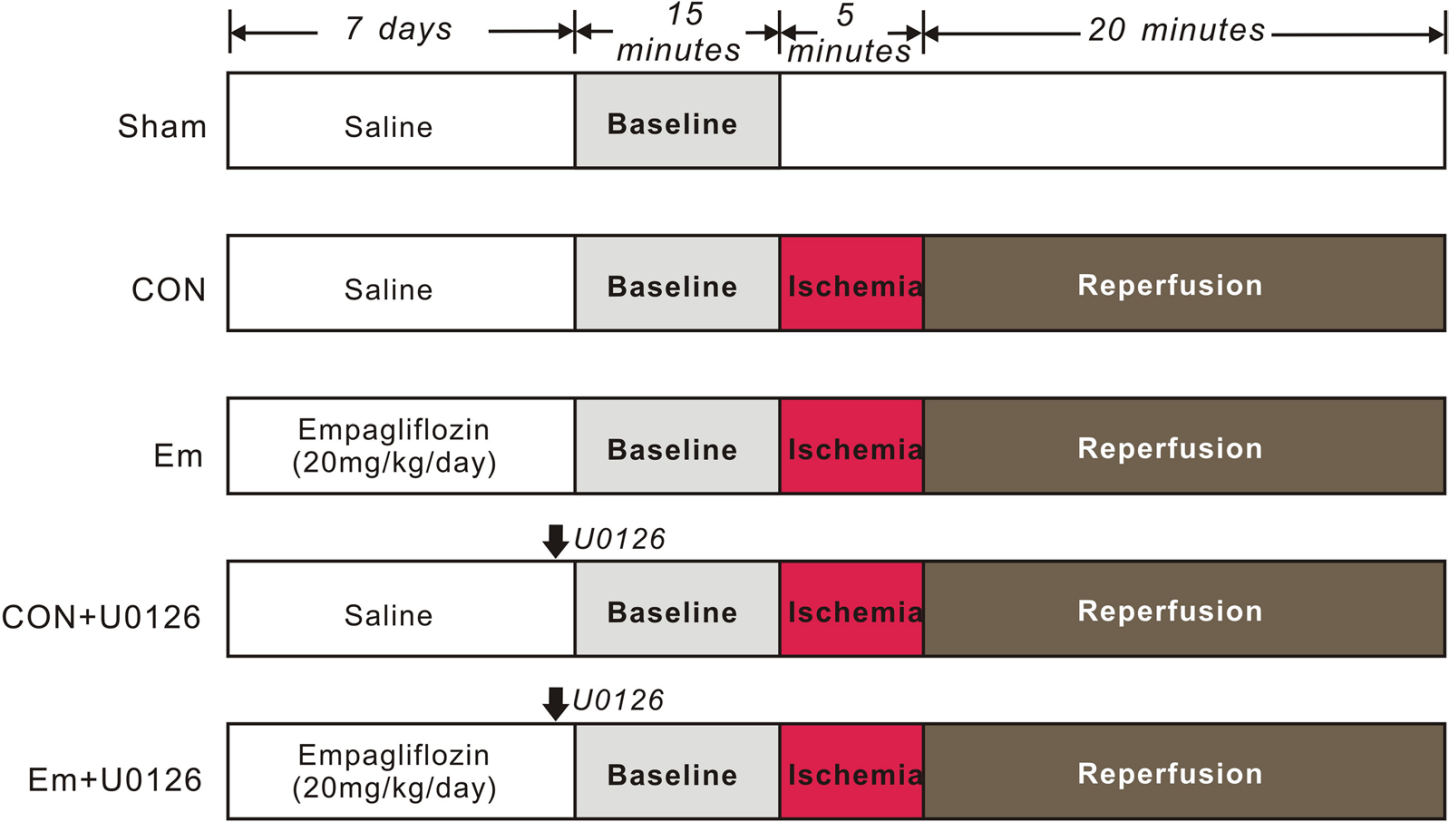
Experimental protocols. All rats except for the sham-operated ones were subjected to cardiac I/R protocol, i.e. 5-min occlusion of the left main coronary artery, followed by 20-min of reperfusion. Rats in Empagliflozin group received 10 mg/kg empagliflozin (dissolved in saline) by intragastric administration once a day for consecutive 7 days. Rats in the control and sham groups were given the same volume of saline. Pharmacological inhibitor (U0126) was administered via the femoral vein as a bolus 30 min prior to myocardial ischemia. Sham, sham-operated group; CON, control; Em, empagliflozin

Animal experiments were performed according to the recommendations in the Guide for the Care and Use of Laboratory Animals of the National Institutes of Health (NIH Publication 8th edition, 2011). The experimental procedures and protocols were approved by the Institutional Animal Care and Use Committee of Sichuan University (Sichuan, China, approval number: 20211201 A). Male Sprague Dawley rats (220–250 g, 7–8 weeks of age) were obtained from Chengdu Dashuo Experimental Animal Research Center (Chengdu, China), and were housed in 12-h light–dark cycles at 20–25 °C, with free access to food and water. The experimental protocol is illustrated in Fig. 1. The rats were divided into five groups: Sham-operated group (sham, n = 5): rats in the sham group received a thoracotomy; the suture was placed under the left main coronary artery but was not tightened. Control group (CON, n = 13): to produce myocardial ischemia/reperfusion injury, rats were administered a 5-min left main coronary artery ligation followed by 20 min reperfusion. Empagliflozin group (Em, n = 15): empagliflozin (Ingelheim am Rhein, Germany) was dissolved in physiological saline (0.9% NaCl) solution and administrated to the rats by oral gavage (20 mg/kg) once a day for 7 days before myocardial ischemia. The dose of empagliflozin was chosen based on previously published studies [8, 10, 11]. On days 1–7, sham and controls received intragastric administration of physiological saline (0.9 % NaCl) alone. To delineate the role of ERK1/2 in empagliflozin-mediated cardioprotection, ERK1/2 inhibitor U0126 (0.5 mg/kg) (Sigma, St. Louis, MO, USA) was intravenously bolus-injected into the femoral vein 30 min prior to myocardial ischemia in control rats (CON + U0126 group, n = 10) or rats treated with empagliflozin (Em + U0126 group, n = 10).

### Surgical procedures

As previous described [12], rats were anesthetized with 50 mg/kg sodium pentobarbital (i.p.) and placed on a heating pad to maintain body temperature. The adequacy of anesthesia was monitored throughout the experiment by observing the absence of toe pinch reflex and corneal reflex. Rats were mechanically ventilated by a rodent ventilator (tidal volume: 8 ml, frequency: 80 times/min, expiration:inspiration:5:4.) (Chengdu Taimeng Technology Co., Ltd., Chengdu, China). After surgical preparation and instrumentation, rats were subjected to 15 min stabilization. All rats received thoracotomy, and then, the main left coronary artery was identified and a 6-0 silk ligature (Ethicon, Somerville, NJ, USA) was placed under the left main coronary artery to perform I/R injury. Coronary artery occlusion was verified by the presence of ventricular dyskinesia and cyanosis in the epicardia. Reperfusion was completed by loosening the snare and confirmed by the appearance of an epicardial hyperemic response. All rats in the experimental groups except for the sham-operated ones were subjected to 5 min left main coronary artery occlusion followed by 20 min of reperfusion. Euthanasia was performed by an overdose of sodium pentobarbital (200 mg/kg,i.p.) at the end of the experiment and death was confirmed by observation of cessation of respiration and cardiac activity.

### Electrocardiography analysis

A standard limb lead II configuration electrocardiographic system was used for monitoring cardiac conduction activity throughout the experiments (Powerlab/8sp system, AD Instruments, Colorado Springs, CO, USA). After anesthesia, the needle electrodes were placed under the skin. Heart rate, RR, PR, QRS, and QT intervals were measured, and corrected QT interval (QTc) was calculated based on Mitchell’s formular [13]: QTc = QT/(RR/100)^1/2^.

### Arrhythmia analysis

ECG was continuously recorded throughout the entire cardiac reperfusion injury period. Arrhythmia parameters were analyzed offline using LabChart7.2.1 software (AD Instruments, Colorado Springs, CO, USA). Arrhythmia events included: (1) cardiac arrhythmia; (2) ventricular tachycardia (VT), (3) polymorphic VT (PVT); (4) sustained ventricular tachycardia over 1 min (SVT); (5) AV block (AVB); (6) sudden cardiac death (SCD); (7) ventricular fibrillation (VF); (8) VT duration; (9) the longest VT episode; (10) the latency to first recorded VT episode.

### Tissue collection

The main left coronary artery was then re-occluded, 1% Evans blue (Sigma, St. Louis, MO, USA) was injected into the left ventricle to label the perfused (not stained) and non-perfused (stained blue) myocardium. Tissues constituting the perfused region (Area at Risk) were then either fixed in 10% buffered formalin for immunohistological assessment or stored in − 80 °C freezer for western blotting analysis.

### Western blotting

Freshly frozen myocardial tissue samples were homogenized in RIPA lysis buffer containing: 50 mM Tris-HCl (pH7.4), 150 mM NaCl, 1% NP-40, 1 mM EDTA, 0.25% sodium deoxycholate, 1X protease inhibitor cocktail (Sigma- Aldrich, St. Louis, MO, USA) and 1X protease inhibitor cocktail (Sigma- Aldrich, St. Louis, MO, USA). The homogenate was centrifuged to extract the total protein. The supernatant was collected. The BCA method was used to determine protein concentration (Pierce, Rockford, IL, USA). Protein lysates were separated using 12% sodium dodecyl sulfate-polyacrylamide gel electrophoresis (SDS-PAGE). The proteins were then transferred onto nitrocellulose blotting Membrane (Pall Corporation, Pensacola, USA) and blocked with 5% non-fat milk for 45 min at room temperature. The blocked membrane was incubated at 4 °C overnight with specific primary antibodies including phosphorylated P38 MAPK (Thr180/Tyr182) (p-P38 MAPK), total P38 MAPK, phosphorylated STAT-3 (Tyr705)(p-STAT3), total STAT-3, phosphorylated STAT-1(Tyr701)(p-STAT1), total STAT-1, phosphorylated STAT-5 (Tyr694)(p-STAT5), total STAT-5, phosphorylated Akt (ser473)(p-AKT), total Akt, phosphorylated glycogen synthase kinase-3β(Ser9) (p-GSK-3β), total GSK-3β, extracellular signal-regulated kinase1/2 (ERK1/2)(Thr202/Tyr204)(p-ERK), and total ERK1/2 (all, 1:1000, Cell Signaling, Danvers, MA, USA). Blots were then incubated with horseradish peroxidase (HRP)-conjugated goat anti-rabbit IgG secondary antibody (1:5000, Bio-Rad, Hercules, CA, USA) for 2 h at room temperature. After 3 washes with PBS containing 0.1% Tween-20 (PBST), the membranes were incubated in ECL substrate solution (Millipore, Billerica, MA, USA) and imaged on an AmershamImager 600 system (GE healthcare, Little Chalfont, UK). Band densities were determined by ImageJ Data Acquisition Software (National Institutes of Health, Bethesda, MD, USA). Relative abundance was obtained by normalizing the density of target phosphorylated protein against total protein.

### Immunohistochemical staining

AAR regions within the left ventricle were identified with the use of Evans blue. The heart tissues were fixed in 10% neutral buffered formalin for 24 h, and then were embedded in paraffin, sectioned at 5 μm thickness. Serial sections of transverse myocardial were deparaffinized. Antigen retrieval was performed at 100 °C temperature for 15 min, and the slices were treated with H_2_O_2_ for 20 min at room temperature. The heart sections were blocked at 37 °C and then were incubated with primary antibody raised against phosphorylated extracellular signal-regulated kinase1/2 (p-ERK1/2) (Thr202/Tyr204) (Affinity bioscience, Cincinnati, OH, USA) overnight at 4 °C. After that, the sections were washed in PBS for 4 times, followed by incubation with secondary antibody. The sections were then incubated in DAB for chromogen staining and counterstained with hematoxylin. The primary antibody was replaced by a phosphate buffer solution as negative control. Images were obtained by an inverted microscope (CAST system, Olympus A/S, Ballerup, Denmark). Ten sub-images were randomly selected for each slide. Brown staining in the cytoplasm of myocyte was recognized as positive expression. Images were analyzed with Image-pro plus software (Media Cybernetics Inc., Carlsbad, CA, USA).

### Statistical analysis

All values were expressed as the mean ± standard deviation (SD). and analyzed with GraphPad Prism version 8.0 (GraphPad, La Jolla, CA, USA). Fisher’s exact test was used to compare the arrhythmogenesis between non-empagliflozin-treated control and empagliflozin-treated rats. Statistical analyses of two independent values including baseline ECG parameters, VT/LVT durations and VT starting time between control and empagliflozin groups were performed by unpaired two-tailed student’s t-test. One-way analysis of variance (ANOVA) was applied for multiple comparisons over three groups. The Levene’s test was used to test homogeneity of variance. If the variance was equal, the Student Newman Keuls test was performed, while the Dunnett’s T3 test was used when variance was not equal. All P values were two-sided. A P value < 0.05 was considered statistically significant.

## Results

### Empagliflozin reduces susceptibility to reperfusion-induced arrhythmias

**Fig. 2:**
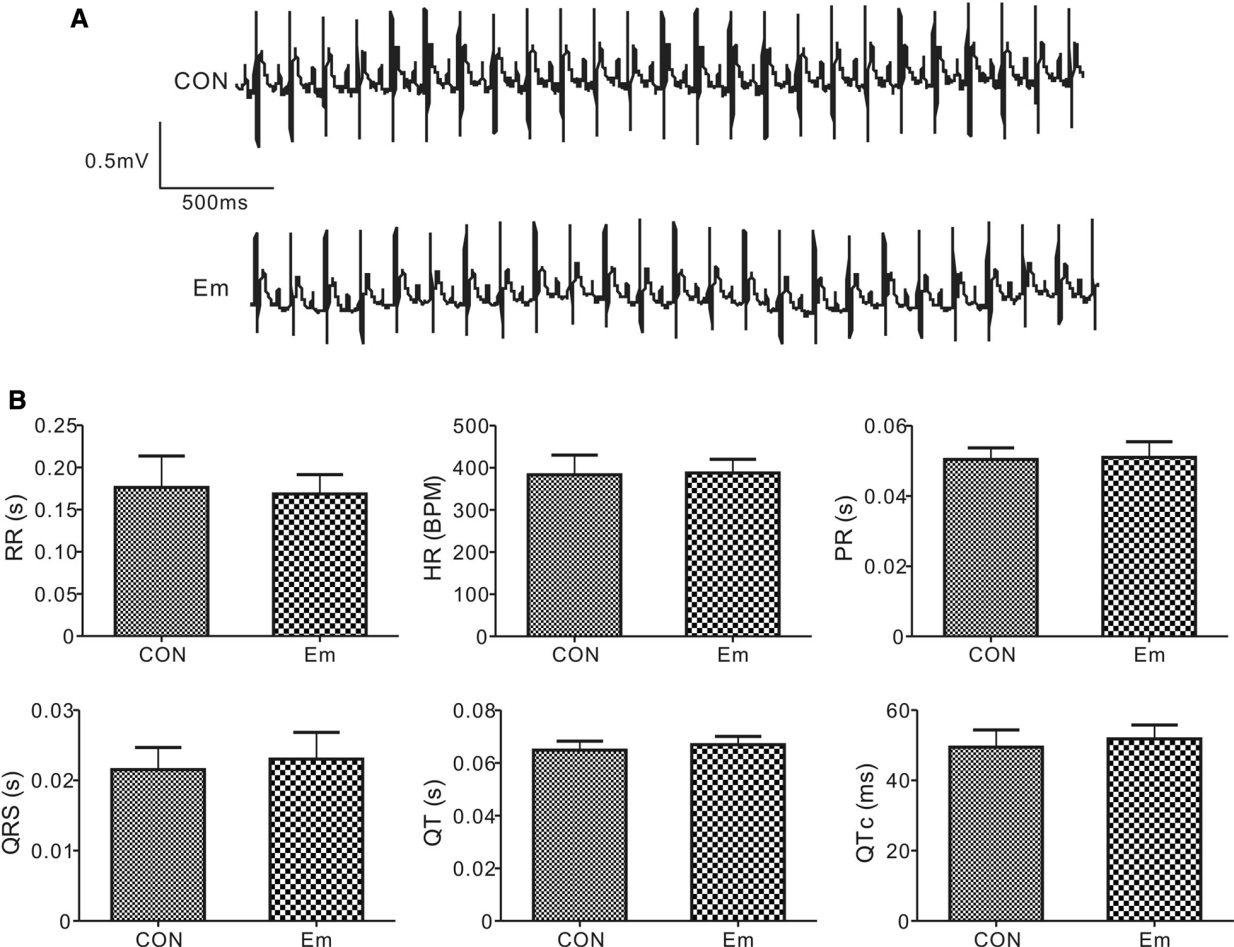
Empagliflozin did not alter baseline ECGs. **A** Exemplar baseline surface ECGs from rats with or without empagliflozin. CON, control; Em, empagliflozin. **B** Quantification of ECG parameters; n = 13–15. All group comparisons, P > 0.05. CON, control; Em, empagliflozin. RR, RR interval; HR, heart rate; PR, PR interval; QRS, QRS interval; QT, QT interval; QTc: QT interval calculated based on Mitchell’s formula (see “Materials and methods”)

**Fig. 3:**
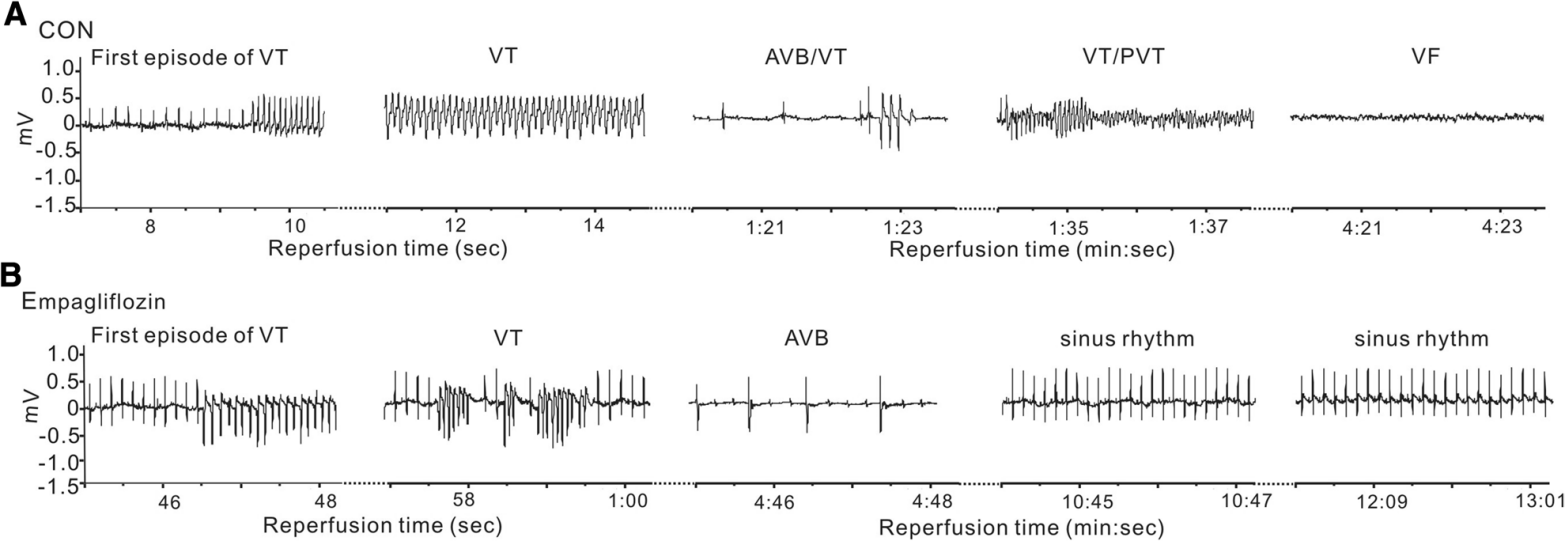
Representative ECG traces during cardiac reperfusion. **A** Representative ECG traces from control rats during the cardiac reperfusion period. CON, control; VT, ventricular tachycardia; First episode of VT, showing the first run of VT; AVB, AV block; PVT, polymorphic VT, VF, ventricular fibrillation.  **B** Representative ECG traces during the cardiac reperfusion period from rats receiving empagliflozin pretreatment

We observed similar electrocardiographic parameters such as heart rate, PR, RR, QRS, QT intervals at baseline in rats with or without empagliflozin treatment, indicating that empagliflozin administration does not affect baseline cardiac normal conduction activity (Fig. 2A, B). We next evaluated the effects of empagliflozin on arrhythmia predisposition upon ischemic stimuli, i.e. main left coronary artery ligation and reperfusion. Figure 3 shows representative ECG tracings from rats with or without empagliflozin after reperfusion injury. The quantification of arrhythmia incidence and mortalities are shown in Table 1. There are several categories of reperfusion-induced arrhythmic events, such as ventricular tachycardia (VT), sustained VT (SVT, VT lasting over 1 min), polymorphic VT (PVT), atrioventricular block (AVB), and sudden cardiac death (SCD). In our study, all 13 control rats (100%) had arrhythmia during the entire reperfusion period; in contrast, only 46.7% rats in empagliflozin group developed arrhythmia (P = 0.0069 vs. CON). Specifically, VT was more prevalent in control rats (13 out of 13, 100%), than in rats in the empagliflozin group (6/15, 40%, P = 0.008). The proportion of sustained VT (> 1 min) incidence was more than 4.6-fold greater in the control group (8/13, 61.5%) than in the empagliflozin group (2/15, 13.3%, P = 0.0163). Half of the control rats (53.8%, 7/13) developed polymorphic VT compared to only 2 out of 15 (13.3%) of the empagliflozin-treated rats (P = 0.0418). Additionally, control rats had severe AV block after cardiac manipulation and the ratio of rats exhibiting AV block was doubled in the control group (11/13, 84.6%) compared to the empagliflozin group (6/15, 40%, P = 0.0238) after cardiac reperfusion.

**Table 1:** Quantification of cardiac arrhythmia incidence

Parameters	CON (n = 13)	Percentage (%)	Empagliflozin (n = 15)	Percentage (%)	P value
Arrhythmia	13	100	7	46.7	0.0069
VT	13	100	6	40	0.0008
SVT (> 1 min)	8	61.5	2	13.3	0.0163
PVT	7	53.8	2	13.3	0.0418
AVB	11	84.6	6	40	0.0238
VF	7	53.8	0	0	0.0014
SCD	9	69.2	0	0	0.0001

VT: ventricular tachycardia (VT); SVT: sustained VT (> 1 min VT); PVT: polymorphic VT; AVB: atrioventricular block; VF: ventricular fibrillation;. SCD: sudden cardiac death

**Fig. 4:**
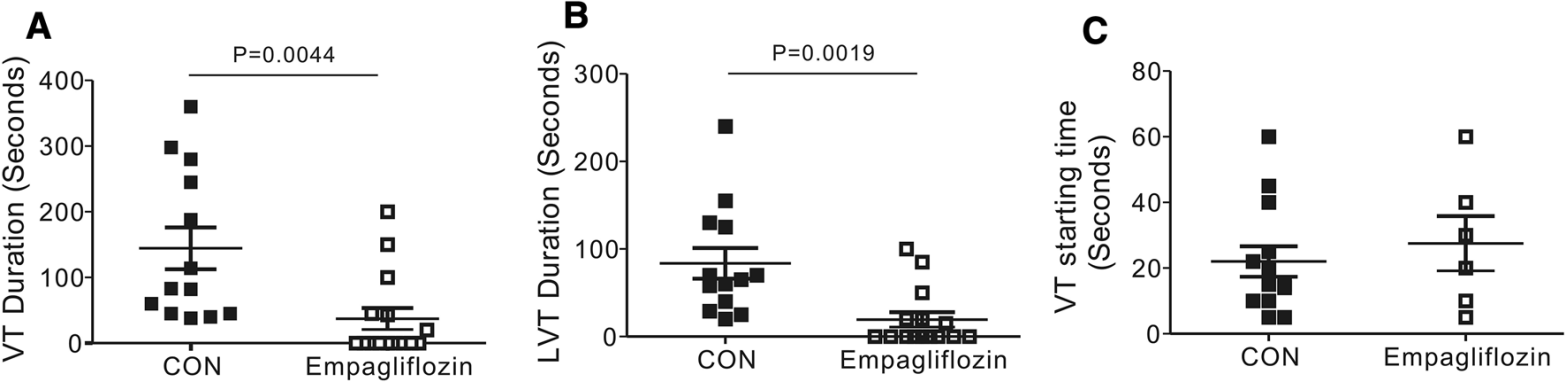
Rats receiving empagliflozin are less predisposed to severe post-IR ventricular tachyarrhythmias. **A** Mean VT durations for rats with or without empagliflozin. n = 13–15. Rats without VT were indicated as 0 s duration. **B** The longest episode of VT duration (LVT) for rats with or without empagliflozin. n = 13–15. **C** The onset time of first VT episode after cardiac reperfusion for rats with or without empagliflozin. n = 13–15

VF occurred frequently in control rats, with 53.8% of the control group exhibiting VF (7 out of 13), while none of the empagliflozin-treated rats that ultimately developed VF (0/15, 0%, P = 0.0014). Death was frequently caused by VF or severe AV block in the control group. Importantly, we found in our study that empagliflozin exerted a powerful protective effect against SCD (zero death/15 empagliflozin-treated rats, compared to 9 deaths/13 control rats; P = 0.0001). Furthermore, during the cardiac reperfusion period, empagliflozin successfully reduced the duration of ventricular arrhythmogenesis; for example, mean VT and the mean longest VT duration were 3.9-fold and 4.3-folder longer, respectively, in control rats compared to empagliflozin-treated ones (mean VT, P = 0.0044, mean LVT, P = 0.0019, Fig. 4A, B). However, empagliflozin did not alter the latency to first recorded VT episode after commencing reperfusion (P > 0.05 vs. CON, Fig. 4C).

### The effect of empagliflozin on post-ischemic signaling pathway induction

**Fig. 5:**
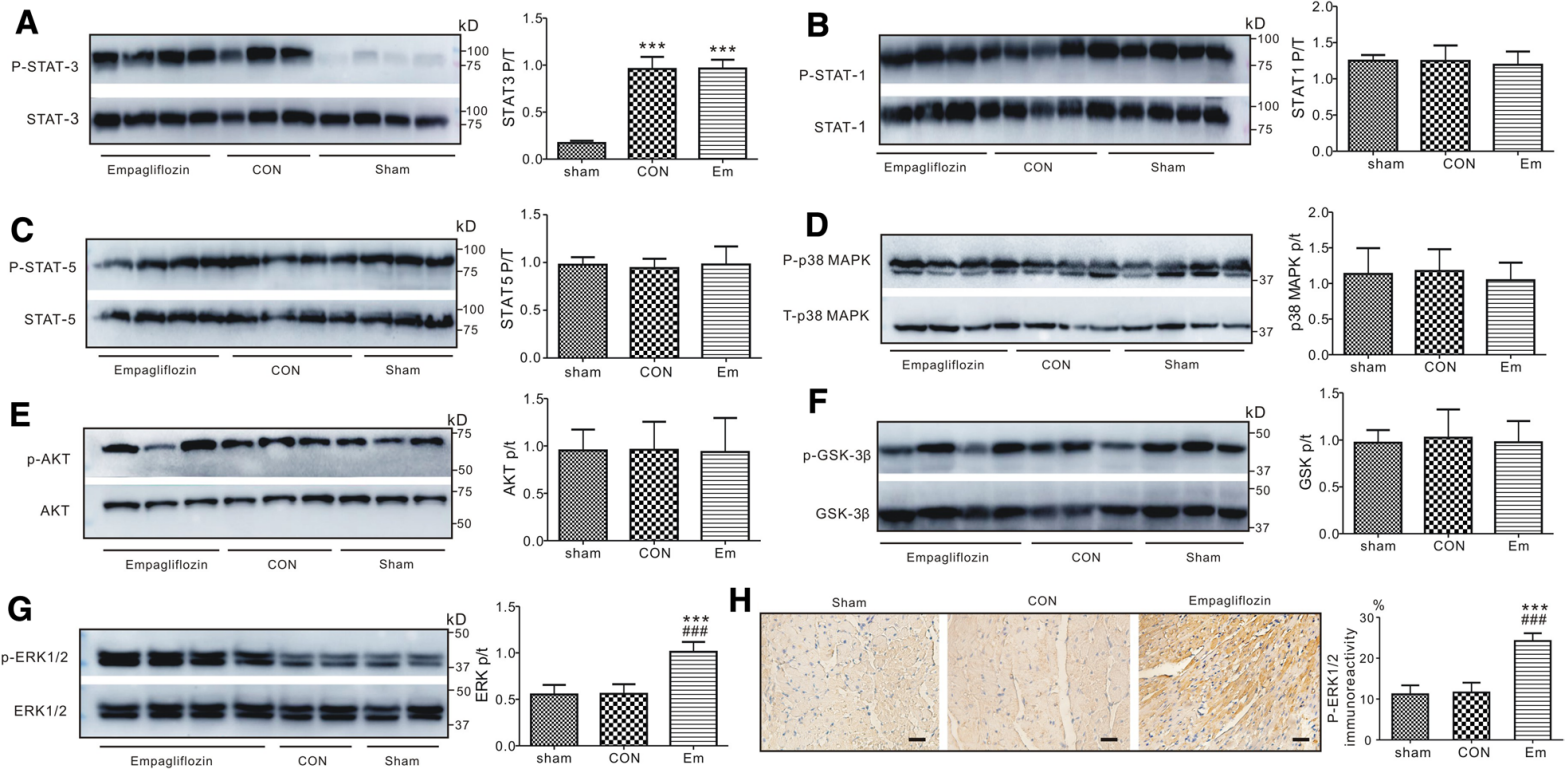
Empagliflozin induces ERK1/2 phosphorylation post cardiac IR injury. Western blots (*Left*) and band densities (*Right*) of phosphorylated STAT3 and total STAT3 (**A**), phosphorylated STAT1 and total STAT1 (**B**), phosphorylated STAT5 and total STAT5 (**C**), ventricular phosphorylated p38 MAPK and total p38 MAPK (**D**), phosphorylated AKT and total AKT (**E**), phosphorylated GSK-3β and total GSK-3β (**F**), phosphorylated ERK1/2 and total ERK1/2 (G), isolated from rats in sham, control and empagliflozin group after 20 min of reperfusion (*n* = 5 per group). ***P < 0.001 vs. sham, ^###^*P < *0.001 vs. CON. Sham, sham-operated group; CON, control; Em, empagliflozin. (H) Left, phosphorylated ERK1/2 immunoreactivity in the cytoplasm of the myocytes of hearts, with and without empagliflozin. ***P < 0.001 vs. sham, ^###^*P < *0.001 vs. CON. Sham, sham-operated group. CON, control. Right, the bar graph illustrates the difference of phosphorylated ERK1/2 expression from each experimental group. Data are presented as mean ± SD. Each group, n = 5

To explore whether or not empagliflozin affects reperfusion-related signaling pathway induction, we studied protein phosphorylation status in the pro-survival reperfusion injury salvage kinase (RISK) pathway, specifically p38 MAPK, ERK1/2, AKT, and GSK-3β; and in the JAK-STAT pathway, specifically STAT-1, STAT-3, and STAT-5. Representative western blots and quantitative results are presented in Fig. 5. The phosphorylation level of each protein was normalized to its corresponding total protein level in each specific signaling pathway. The total protein level of each protein, i.e., STAT-3, STAT-1, STAT-5, p38 MAPK, AKT, GSK-3β, and ERK1/2,was not different in the three groups tested.

Although reperfusion injury caused  >  fivefold greater STAT3 phosphorylation in control and empagliflozin-treated ventricles when compared to sham-operated ones, there was no difference in the expression of phosphorylated STAT3 (Fig. 5A), STAT1 (Fig. 5B) and STAT5 (Fig. 5C) in the ventricles after empagliflozin treatment when compared with controls, indicating that JAK-STAT pathway may not be involved in this empagliflozin-induced anti-arrhythmic activity. Furthermore, we did not detect the differences in phosphorylated p38 MAPK (Fig. 5D), AKT (Fig. 5E) and GSK-3β (Fig. 5F) protein levels among groups after 20 min of reperfusion; therefore, the protective effect offered by empagliflozin was unlikely to arise from changes in p38 MAPK/AKT/GSK-3β signaling molecules in RISK pathway.

Notably, although no differences were detected between sham-operated and control rats, pretreatment with empagliflozin markedly changed phosphorylation of ventricular ERK1/2 (Thr202/Tyr204) (Fig. 5G, P < 0.001), increasing the ratio of phosphorylated (p) to total (t) ERK1/2 to almost double that of sham-operated or control rats following cardiac reperfusion. Meanwhile, phosphorylated ERK1/2 immunoreactivity was also detected in the left ventricular area at risk in each group. In consistent with our western blotting result, we found that p-ERK was similarly phosphorylated in control and sham-operated hearts (P > 0.05); however, the positive expression of p-ERK1/2 protein was significantly increased in the empagliflozin-treated rats versus the sham-operated or control rats (Fig. 5H, P < 0.001).

### Pharmacological inhibition of ERK1/2 impairs the anti-arrhythmic action of empagliflozin

**Fig. 6:**
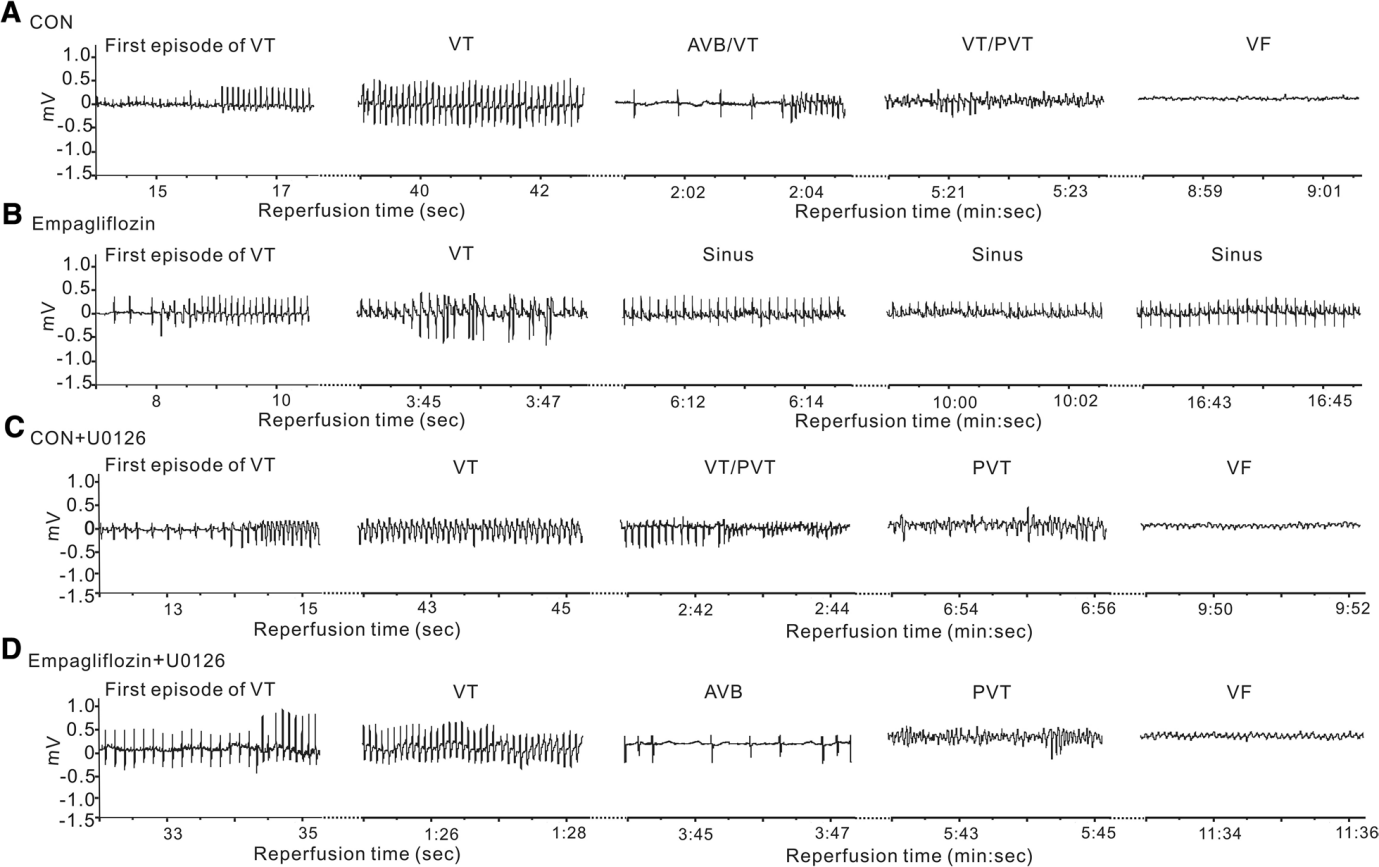
The influence of pharmacological inhibitor on ECGs during cardiac reperfusion. Representative ECG traces from rats in control (**A**), empagliflozin (**B**), control with U0126 (**C**), and empagliflozin with U0126 (**D**) groups during the cardiac reperfusion period. CON, control; VT, ventricular tachycardia; First episode of VT, showing the first run of VT; AVB, AV block; PVT, polymorphic VT, VF, ventricular fibrillation, Sinus, rat in sinus rhythm

**Fig. 7:**
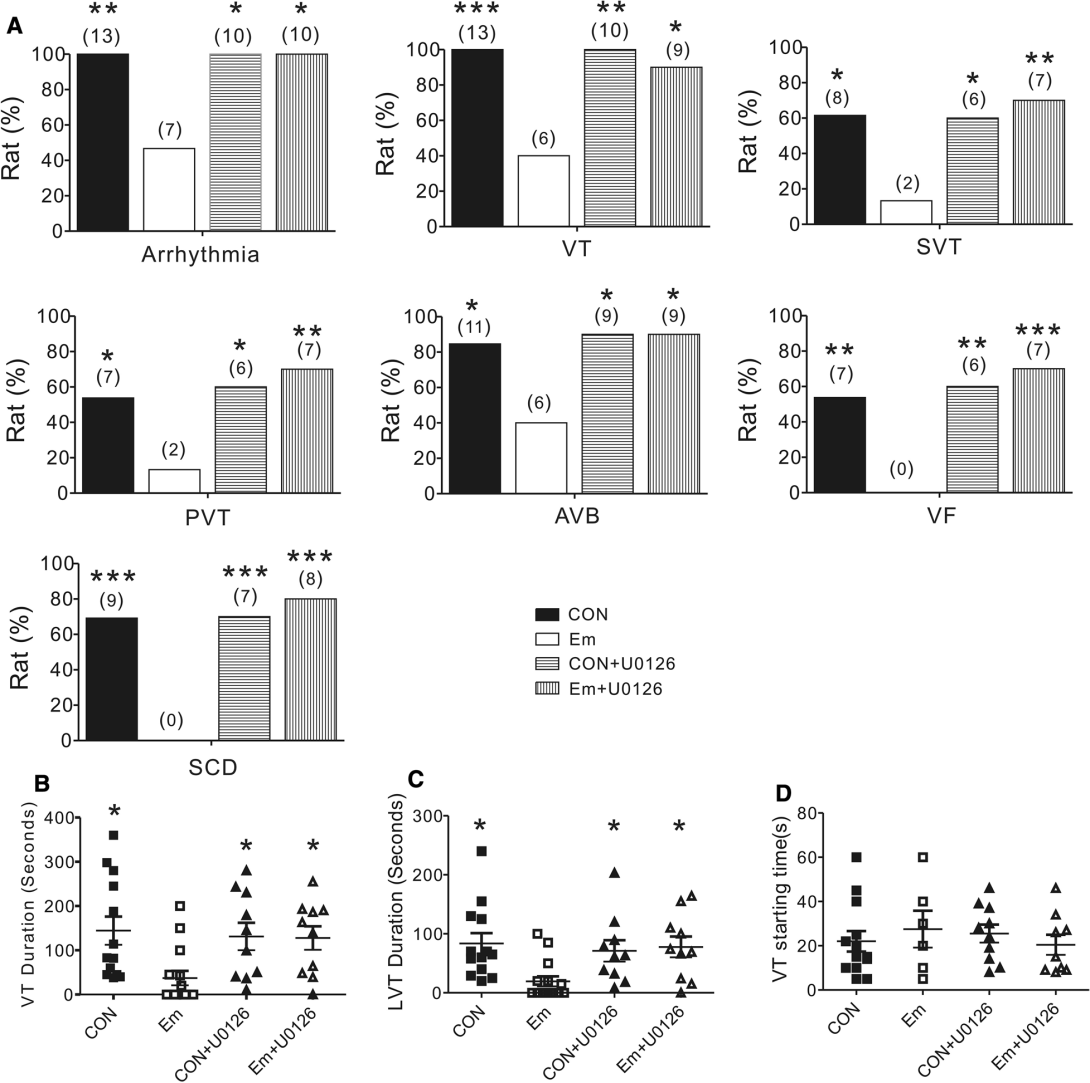
The effect of ERK inhibitor U0126 on post-reperfusion arrhythmia characteristics and mortality. (**A**) Quantification of the incidence of cardiac arrhythmia characteristics and mortality during post-ischemia reperfusion in rats with myocardial I/R injury. Numbers of animals per category are indicated in parentheses. CON, control; Em, empagliflozin; VT, ventricular tachycardia; First episode of VT, showing the first run of VT; AVB, AV block; PVT, polymorphic VT, VF, ventricular fibrillation, Sinus, rat in sinus rhythm. *P < 0.05, **P < 0.01 and ***P < 0.001 vs. Em. Each group included 10–15 rats. Values for control and rats with empagliflozin are repeated from Table 1 for comparison. (**B**) Mean VT durations in the four study group. Rats without VT were indicated as 0 s duration. Each group included 10–15 rats. Values for control and rats with empagliflozin are repeated from Fig. 4 for comparison. (**C**) The longest episode of VT duration (LVT) in the four study group. Rats without VT were indicated as 0 s duration. Each group included 10–15 rats. Values for control and rats with empagliflozin are repeated from Fig. 4 for comparison. (**D**) The onset time of first VT episode after cardiac reperfusion in the four study group. Each group included 10–15 rats. Values for control and rats with empagliflozin are repeated from Fig. 4 for comparison

As ERK1/2 phosphorylation was increased after reperfusion injury in empagliflozin-treated rats, to further determine the role of ERK1/2 in this empagliflozin-induced cardioprotection, we next applied U0126, a pharmacological inhibitor of ERK1/2, prior to cardiac I/R. Typical electrocardiographic recordings are shown in Fig. 6A–D. Strikingly, while U0126 had no effect on post-reperfusion arrhythmia incidence in control rats (Fig. 6A,C), U0126 completely reversed the empagliflozin-dependent anti-arrhythmic effect (Fig. 6B,D). Post-I/R arrhythmia incidence and severity were largely increased after U0126 administration in the empagliflozin group. For example, after U0126 application, all rats in the empagliflozin group developed arrhythmia. Specifically, 90% (9/10), 70% (7/10), 70% (7/10), 90% (9/10), 70% (7/10) of empagliflozin-treated rats with U0126 developed VT, SVT, PVT, AVB, or VF, respectively, with an increased incidence (80%, 8/10) of SCD during reperfusion as compared with empagliflozin-treated rats without U0126 (0%, 0/15) (P < 0.05, P < 0.01, or P < 0.001 Fig. 7A). In the meantime, U0126 caused significant prolongation of both mean VT duration and the mean longest VT episode in rats pretreated with empagliflozin, to levels similar to those of control group rats without empagliflozin (P < 0.05 vs. empagliflozin-treated rats, Fig. 7B, C), while the latency to the first VT episode was similar in either group (P > 0.05, Fig. 7D).

### The cardioprotective effects of empagliflozin are mediated by an ERK-dependent signaling pathway

**Fig. 8:**
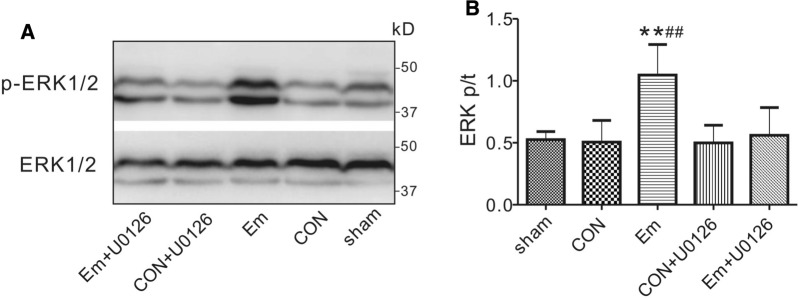
The influence of U0126 on ventricular ERK1/2 phosphorylation. Western blots (**A**) and band densities (**B**) of ventricular phosphorylated ERK1/2 and total ERK1/2 in control and empagliflozin groups in the presence or absence of pharmacological inhibitor after 20 min of reperfusion (*n* = 5 per group). **P < 0.01 vs. sham, ^##^*P < *0.01 vs. CON. Sham, sham-operated group; CON, control; Em, empagliflozin

We also tested the effects of ERK1/2 inhibitor U0126 on ERK1/2 protein phosphorylation. ERK1/2 phosphorylation was impaired by U0126, which halved the ERK1/2 phosphorylation level from 1.05 ± 0.25 (empagliflozin) to 0.56 ± 0.22 (empagliflozin + U0126) arbitrary units (*P < *0.01, vs. sham-operated rats, or *P < *0.01, vs. control rats). Meanwhile, there was no difference between U0126-treated control and U0126-treated empagliflozin rats with respect to the phosphorylation level of ERK1/2 after reperfusion (Fig. 8A,B).

## Discussion

### Reperfusion and sudden cardiac death

Cardiovascular disease (CVD) is a leading cause of morbidity and mortality worldwide. CVD takes an estimated 17.9 million lives annually, accounting for 31% of all global deaths according to World Health Organization (WHO) estimates. Ischemic heart disease is the most common type of CVD. Over 750 thousand individuals suffer myocardial infarction in the United States alone annually [14]. Without promptly effective therapeutic intervention such as coronary artery recanalization and myocardial reperfusion, the ischemic insult will cause permanent damage and cell necrosis in the heart [15]. The standard reperfusion strategy used in acute coronary syndrome may salvage the ischemic myocardium, yet also acts as a “double-edged sword” to the heart, inducing ischemia-reperfusion injury. Reperfusion may therefore worsen myocardial damage, decrease ventricular function and elicit further pathologic consequences. In particular, it can lead to life-threatening ventricular arrhythmias and, ultimately, sudden cardiac death [1]. Therefore, identifying therapeutic targets and optimizing the treatment of patients at high risk of death post-myocardial infarction are of great importance.

Numerous experimental studies have therefore been focused upon finding protective strategies or elucidating underlying arrhythmogenic mechanisms, with the rat being a popular model system. Sakamoto et al. found that the relationship between ischemia duration and incidence of reperfusion-induced lethal arrhythmia was “bell shaped” in rats, i.e., a maximum of 5 min of myocardial ischemia resulted in the highest incidence of VF upon reperfusion [16]. Moreover, other studies showed that ventricular arrhythmias can be provoked within the first 20 min of reperfusion in rat heart [17]. Therefore, in the present study, we used an ischemia-reperfusion model of acute myocardial injury in rats, incorporating a 5-min reversible ligation of the left coronary artery to provoke ventricular tachycardia and fibrillation. The present study is the first to characterize the anti-arrhythmic activity of the SGLT2 inhibitor and T2DM medication, empagliflozin, on ischemia-provoked severe ventricular arrhythmias in vivo. We found that 100% of control rats had VT, 53.8% exhibited VF (7 /13) and 69.2% (9/13) of them suffered SCD during the entire 20-min of reperfusion. Our model replicates reperfusion-induced arrhythmia during acute myocardial infarction, which is of clinical relevance but does not incorporate the chronic cardiac remodeling after acute myocardial infarction that may also affect reperfusion-induced arrhythmogenesis.

### SGLT2 inhibitors and cardioprotection

T2DM is a chronic metabolic disease that affects 415 million people worldwide [18]. Over 32% patients with T2DM have cardiovascular disease [19]. SGLT2 inhibitors are a recently developed novel class of antidiabetic drugs that inhibit glucose and sodium reabsorption from the proximal tubule of the kidney. There are four main SGLT2 inhibitors in clinical use, canagliflozin, dapagliflozin, empagliflozin, and ertugliflozin. Several major clinical cardiovascular outcome trials, such as the EMPA-REG outcome trial [2], CANVAS trial [20], DECLARE-TIMI 58 trial [21], and VERTIS-CV trial [22] have all shown that SGLT2 inhibitors can significantly reduce the risk of cardiovascular complications, cardiovascular mortality and heart failure hospitalizations in T2DM patients. The follow-up meta-analysis also supports these results and suggests that SGLT2 inhibitors are associated with reduced risks of incident atrial arrhythmias and sudden cardiac death in patients with T2DM [23]. More importantly, these beneficial cardioprotective effects have now been shown to extend to non-T2DM patients [24]. Results from the EMPEROR-Reduced trial [25] and the DAPA-HF trial [26] suggested that empagliflozin and dapagliflozin can improve cardiac outcomes among non-T2DM patients with heart failure with reduced ejection fraction (HFrEF) [25]. Consistent with results from these clinical trials, Requena-Ibáñez et al. provided further evidence that empagliflozin improves adiposity, interstitial myocardial fibrosis, aortic stiffness, and inflammatory markers in nondiabetic patients with HFrEF [27]. Moreover, the final data from the EMPEROR-Preserved Phase III trial indicate that empagliflozin significantly reduces the risk of the composite of cardiovascular death or hospitalization for heart failure patients with preserved ejection fraction (HFpEF), with or without T2DM, indicating that the cardiovascular benefits of SGLT2 inhibitors are unlikely to be associated with improved glycemic control [3].

Accumulating experimental evidence has confirmed the benefits of SGLT2 inhibitors in various animal models, showing their favorable role in reducing the development and progression of cardiovascular diseases. For example, the cardioprotective effects of empagliflozin were attributed to the alleviation of atrial remodeling in high-fat diet/streptozotocin-induced diabetic rats [8], improvement of myocardial energetics in non-diabetic heart failure pigs [28], prevention of cardiac remodeling, endothelial dysfunction and attenuation of cardiac fibrosis in the metabolic syndrome ZSF1 rat [5], non-diabetic post-infarct rats with either LV dysfunction [29] or heart failure [30], and also in hypertensive heart failure rats [6].

Focusing on acute cardiac I/R injury, recent reports also demonstrated that empagliflozin [7] or dapagliflozin [31] markedly limited infarct size and improved LV function post I/R in non-diabetic rodents. Our results confirm and extend these recent reports by demonstrating that empagliflozin offers strong anti-arrhythmic effects against myocardial ischemia/reperfusion injury in vivo, as seen by significantly reducing myocardial vulnerability to sudden cardiac death and all types of post-I/R arrhythmias, such as reducing incidence of VT, SVT, PVT, AVB and VF, and reducing VT and LVT durations post I/R injury. Notably, we showed that empagliflozin did not change the latency to the first recorded VT episode after commencing reperfusion. In contrast, using a different protocol, Lahnwong and colleagues showed that rats pretreated with 1 mg/kg dapagliflozin had a delayed time to first VT/VF onset [31]. The discrepancy may result from differences in the medication, the administration protocol, the animal disease model and/or the experimental design. Meanwhile, it is worth mentioning that we used a non-diabetic rat model of myocardial I/R injury, so the results we obtained may provide experimental evidence for SGLT2 inhibitor therapies for non-T2DM patients with acute coronary syndrome. In the meantime, in addition to its approval for treating T2DM, empagliflozin has earned a new indication from the FDA for reduction of the risk of cardiovascular death in patients with T2DM and cardiovascular disease [32]. Our investigations may also give clues for optimizing current strategies of revascularization therapy in individuals with T2DM and cardiovascular disease, to reduce perioperative and postoperative complications associated with myocardial I/R injury.

### Cardioprotective mechanisms

Although the above-mentioned beneficial effect of empagliflozin in the presence or absence of T2DM has been confirmed by both clinical and experimental studies, its underlying molecular mechanism is still in the exploratory stage. Moreover, the possible mechanisms of empagliflozin-induced anti-arrhythmic effects after acute myocardial ischemia are unclear. Intensive analysis of the underlying mechanisms for cardioprotection against reperfusion, especially the signaling pathways, has identified major pro-survival pathways, the JAK-STAT pathway and the reperfusion injury salvage kinase (RISK) pathway, both of which transmit cardioprotective signals from the sarcolemmal caveolae to the mitochondria.

The JAK/STAT pathway, originally discovered as a cytokine-stimulated signal transduction pathway [33], is now known to play a crucial role in myocardial I/R injury [34]. STATs, including the STAT1-6 subfamily, are a unique class of transcription factors in the JAK-STAT pathway. It has been shown that the activation of STATs is associated with multiple cardioprotective strategies; however, the inhibition of STAT phosphorylation by the JAK inhibitor AG-490 blocked cardioprotection offered by ischemia preconditioning or postconditioning [35]. We have uncovered dissociation between JAK-STAT pathway activation, specifically STAT1, STAT3 and STAT5, and the anti-arrhythmic effects of empagliflozin in the *in vivo* rat heart, indicating that there may be an alternative prosurvival signal transduction pathway responsible for the protection of the myocardium against lethal reperfusion-induced arrhythmia.

The concept of a cardioprotective RISK pathway was first proposed by the Yellon group [36]. p38 mitogen-activated protein kinase (p38MAPK), protein kinase B (AKT), and extracellular signal-regulated kinases (ERK1/2) are vital signaling molecules in this antiapoptotic reperfusion pathway. Glycogen synthase kinase-3β (GSK-3β) serves as the major downstream target of RISK pathway [37]. Preclinical studies have confirmed that cardioprotective agents or therapies, such as adipocytokines, adenosine, volatile anesthetics or ischemic conditioning have infarct-sparing effects after reperfusion injury through the activation of the RISK pathway [38]. Meanwhile, our previously published data are consistent with these earlier studies, as we found that remote ischemic liver preconditioning enhanced ERK1/2 and GSK-3β Ser9 phosphorylation, while diminishing predisposition to lethal arrhythmias after reperfusion [39]. In the current study, using a 5-min period of ischemia followed by 20-min of reperfusion protocol, we were able to demonstrate that pretreatment with empagliflozin prior to myocardial ischemia reduced the incidence and severity of reperfusion-induced arrhythmogenesis and increased ERK1/2 phosphorylation (activation) post-I/R. Importantly, pharmacological inhibition of empagliflozin-induced ERK1/2 activation abolished the anti-arrhythmic effects of empagliflozin and abrogated the phosphorylation of ERK1/2. Our experimental study has demonstrated directly the involvement of empagliflozin in recruiting ERK1/2 in the RISK pathway to protect the heart against lethal myocardial reperfusion injury.

## Limitations

We acknowledge potential limitations of this study. First, the dosing of empagliflozin in clinical trials (10 mg or 25 mg/day [2], equivalent to no more than 3 mg/kg/day for rats) is lower than what has been established in our current study using a rat model (20 mg/kg/day for 7 days). However, In other preclinical studies, empagliflozin provided cardioprotection with a reported dose range of 10–30 mg/kg/day in rodents [5, 7, 8, 11]. In line with these studies, we showed that empagliflozin successfully reduced the incidence of all types of arrhythmia after reperfusion. Furthermore, identical experimental protocols were adopted in each group; therefore, by extrapolation, we conclude that empagliflozin acts to protect the heart against reperfusion injury-induced lethal arrhythmia. Second, we only observed the possible anti-arrhythmic effect of empagliflozin on several reperfusion-related signaling pathways; further experiments are needed to expand knowledge of its underlying mechanisms in term of signal transduction and address potential cross-talk between signaling molecules and pathways. Third, to avoid the possible influence of volatile anesthetics, which are known to mediate cardioprotection [40], we anesthetized all rats with sodium pentobarbital. By using an identical anesthetic protocol between groups, we showed that empagliflozin exerted a powerful protective effect against SCD after cardiac reperfusion. Fourth, the primary goal of our study was to determine whether empagliflozin is cardioprotective in the context of reperfusion-induced cardiac arrhythmias in non-T2DM rats. We did not evaluate its effect in T2DM rats, and therefore it is worth exploring the anti-arrhythmic effect of empagliflozin in hyperglycemic individuals in the future. Furthermore, we only assessed the anti-arrhythmic effect of empagliflozin in our study; the functional importance of other SGLT2 inhibitors, such as dapagliflozin in reperfusion-induced arrhythmia, therefore remains to be elucidated.

## Conclusions

In summary, our data confirm benefits of empagliflozin in terms of reducing predisposition to lethal arrhythmia and sudden cardiac death induced by reperfusion injury after acute myocardial infarction. Furthermore, this empagliflozin-induced cardioprotection is independent from glucose control and is associated with the ERK1/2-dependent cell-survival signaling pathway.

## Data Availability

All data generated or analyzed during this study are included in this published article.

## Abbreviations

SGLT2: Sodium–glucose cotransporter 2
SCD: Sudden cardiac death
CABG: Coronary artery bypass grafting
PCI: Percutaneous coronary intervention
RISK: Reperfusion injury salvage kinase
GSK-3β: Glycogen synthase kinase-3β
STAT: Signal transducer and activator of transcription
JAK: Janus kinase
ERK1/2: Extracellular signal-regulated kinases 1 and 2
AKT: Protein kinase B
AAR: Area at risk
T2DM: Type 2 diabetes mellitus
HFpEF: Heat failure patients with preserved ejection fraction
VT: Ventricular tachycardia
PVT: Polymorphic ventricular tachycardia
SVT: Sustained ventricular tachycardia over 1 minute
AVB: Atrioventricular block
